# New paste for severe stomatitis in patients undergoing head-and-neck cancer radiotherapy and/or chemotherapy with oral appliance

**DOI:** 10.1186/s12885-018-4017-2

**Published:** 2018-03-02

**Authors:** Ayumi Sakuramoto, Yoko Hasegawa, Kazuma Sugahara, Yoshiyuki Komoda, Kana Hasegawa, Shinichi Hikasa, Mai Kurashita, Junya Sakai, Masahiro Arita, Kazuhiro Yasukawa, Hiromitsu Kishimoto

**Affiliations:** 1 Department of Dentistry and Oral Surgery, Hyogo College of Medicine, 1-1 Mukogawa-cho, Nishinomiya, Hyogo 663-8501 Japan; 2 Department of Chemical Science and Engineering, Graduate School of Engineering, Kobe University, Kobe, Hyogo 657-8501 Japan; 3 Department of Pharmacy, Hyogo College of Medicine, 1-1 Mukogawa-cho, Nishinomiya, Hyogo 663-8501 Japan; 4 Division of Occlusion & Maxillofacial Reconstruction, Department of Oral Function, School of Dentistry, Kyushu Dental University, Kitakyushu, Fukuoka 803-8580 Japan; 5 Medical Research Group, Development Department. Takiron Co., Ltd., Osaka, Japan

**Keywords:** Stomatitis, Head-and-neck cancer, Treatment paste, Denture adhesives, Radiotherapy and/or chemotherapy

## Abstract

**Background:**

The aim of the present study was to evaluate the physical properties of “admixture paste”, which is a commercially available gel containing hinokitiol for use against severe stomatitis, and its characteristics as a moisturizing gel and denture adhesive.

**Methods:**

The admixture paste, which contained dexamethasone (Dexaltin®), gel for oral care (Refrecare H®) and petrolatum, and its 3 components, either alone or in different combinations, were subjected to viscosity, adhesiveness and elution testing to compare their physical properties. Viscosity was measured with a stress-controlled rheometer. Adhesive force was measured by tension test. Elution under a simulated oral environment was evaluated by monitoring with a fixed-point camera and absorbance. Both adhesiveness and elution were evaluated every hour for 6 h. A linear mixed-effects model was used to assess differences in the time course of elution between samples. In 3 og-rank test was used to compare time to elution into saliva among samples.

**Results:**

The results of viscosity testing demonstrated that the admixture paste had similar viscosity to cream-type denture adhesives and this was temperature independent. In the adhesiveness tests, the admixture paste showed stronger adhesiveness than that of cream-type denture adhesives. In the elution test, the admixture paste demonstrated gradual dissolution and apparent temporal changes for 6 h in a simulated oral environment.

**Conclusions:**

The results of the present study demonstrated that the admixture paste has adhesive force similar to those of denture adhesives and good local retention in saliva, and that it might be suitable for therapeutic use in patients with severe stomatitis derived from radiotherapy and/or chemotherapy for cancer.

**Electronic supplementary material:**

The online version of this article (10.1186/s12885-018-4017-2) contains supplementary material, which is available to authorized users.

## Background

Multidisciplinary treatments consisting of surgery, radiotherapy and chemotherapy are performed for patients with malignancy [1]. However, these treatments can induce severe oral stomatitis, thus interfering with oral ingestion. In our daily clinical practice, we frequently encounter patients undergoing treatment by radiotherapy and/or chemotherapy (hereinafter referred to as “CRT”) for cancer in the head and neck region with severe oral stomatitis. Radiotherapy for head and neck cancer has a nearly 100% risk of causing oral stomatitis in the irradiated area [2, 3]. Chemotherapy is also associated with a 1–10% risk of causing severe oral stomatitis [4, 5]. However, there is no established pre-treatment management practice for preventing stomatitis, as the method of treatment for stomatitis varies between institutions [6].

At the Department of Dentistry and Oral Surgery in the College of Medicine, we provide pre-operative oral management for almost all patients with head and neck cancer among those who are hospitalized. For stomatitis occurring in these patients after CRT, we perform oral management practices, such as oral cleaning, application of gel for oral care, laser treatment and use of mouthwash containing local anesthetic. Many of the patients with head and neck cancer are elderly and have postoperative defects of teeth, jaw bone and oral tissue, which is necessary for the use of oral appliances such as removable dentures and palatal augmentation prosthesis (PAP) [7]. Thus, there are many patients who experience severe stomatitis including mucositis, which interferes with the use of dentures during CRT. The interruption of oral appliance use significantly affects quality of life (QOL) by preventing patients from talking and eating [8–10]. Severe stomatitis may result in interruption or even discontinuation of cancer treatment [11] by infections derived from severe oral stomatitis, neutropenia and/or uncontrollable pain. Nevertheless, no established treatment is currently available for severe stomatitis in patients undergoing head-and-neck cancer CRT with oral appliances. Dexamethasone ointment and other steroid ointments are often prescribed for the purpose of pain relief, but indiscriminate use of these agents may induce microbial substitution with *Candida* and other fungi [12, 13]. Furthermore, steroid ointments are effective against oral stomatitis caused by head-and-neck cancer treatments such as CRT [14]. Gel for oral care is often prescribed for pain relief and to moisten the oral cavity. In general treatment, all of these components are used separately, but not simultaneously.

In our clinical practice, we found that dexamethasone and oral gel with hinokitiol are effective when mixed together because the viscosity is increased. Vaseline is added to further adjust the viscosity, which improves the handling. Subsequently, we mixed various ointments and oral gels for use against oral stomatitis with the aim of finding a paste having an appropriate coefficient of viscosity. As a consequence, we developed an “admixture paste” formulated from equal amounts of dexamethasone ointment (Dexaltin® Oral Ointment, Nippon Kayaku Co., Ltd., Tokyo, Japan; hereinafter referred to as “Dexi”), gel for oral care (Refrecare® H, Nippon Zettoc Co., Ltd., Tokyo, Japan; “Moist”) and petrolatum (Kenei Seiyaku, Osaka, Japan; “Vase”) for infection prevention and symptom relief of severe stomatitis in patients with head and neck cancer. With this paste, we expect good local retention and lasting drug/gel efficacy regardless of the flow of saliva.

For clinical use of “admixture paste”, we obtained approval from the Hyogo College of Medicine Ethics Committee regarding the safety and approach used. Subsequently, the admixture paste was available for short-term (maximal 3 weeks) treatment of severe stomatitis in patients undergoing head-and-neck cancer CRT and using oral appliances (dentures and PAP). Short-term use of the admixture paste for head and neck cancer with severe stomatitis resulted in symptom relief of severe stomatitis and appeared to provide stability of oral appliances. We have not yet performed quantitative assessment of the physical properties (viscosity and elution characteristics to saliva) of this admixture paste.

The aim of the study was to evaluate the physical properties and moisturizing characteristics of “admixture paste” (containing dexamethasone, gel for oral care and petrolatum), which has a viscosity and adhesiveness equivalent to that of denture adhesive.

## Methods

### Preparation of mixed paste

The components of the admixture paste and their compositions are shown in Table 1. The admixture paste was prepared by mixing equal volumes of Dexi, Moist and Vase in a rubber cup for dental use (28 mm in inner diameter and 33 mm in height; Tokuyama Dental, Osaka, Japan) using a metal spatula (YDM Corporation, Tokyo, Japan) for 30 s until a homogeneous knead was obtained. Kneading was performed by either a dentist or a dental hygienist. The obtained admixture paste was stored in an airtight container at room temperature for up to 24 h before use. An additional movie file shows the paste preparation in more detail (see Additional file 1).

**Table 1:** Composition of each material

Dexi		Moist		Vase	
Active component	Dexamethasone	Active component	Hinokitiol Dipotassium glycyrrhizinate	Active component	White vaseline
Additive	Liquid paraffin Sodium polyacrylate Plastibase	Sweetener	Xylitol		
		Solubilizing agent	Polyoxyethylene hydrogenated castor oil		
		Preservative	Sodium benzoate		
		Preservative	A hydrogenphosphate melanian snail thorium Citric acid Humecant sodium hyaluronate (2) Concentrated glycerin A propylene glycol		
		Solvent	Purified water Ethanol		
		Binding agent	Sodium polyacrylate A carrageenan		
		Stabilizer	An edetic acid melanian snail thorium		
		Flavor	Rifrecare H and menthol		

Dexi: dexamethasone ointment (Dexaltin® Oral Ointment); Moist: gel for oral care (Refrecare H®); Vase: petrolatum


**Additional file 1:** Mixing of three components to make the admixture paste. (3GP 1.19 MB)


### Physical property evaluation

#### Viscosity measurement

**Fig. 1:**
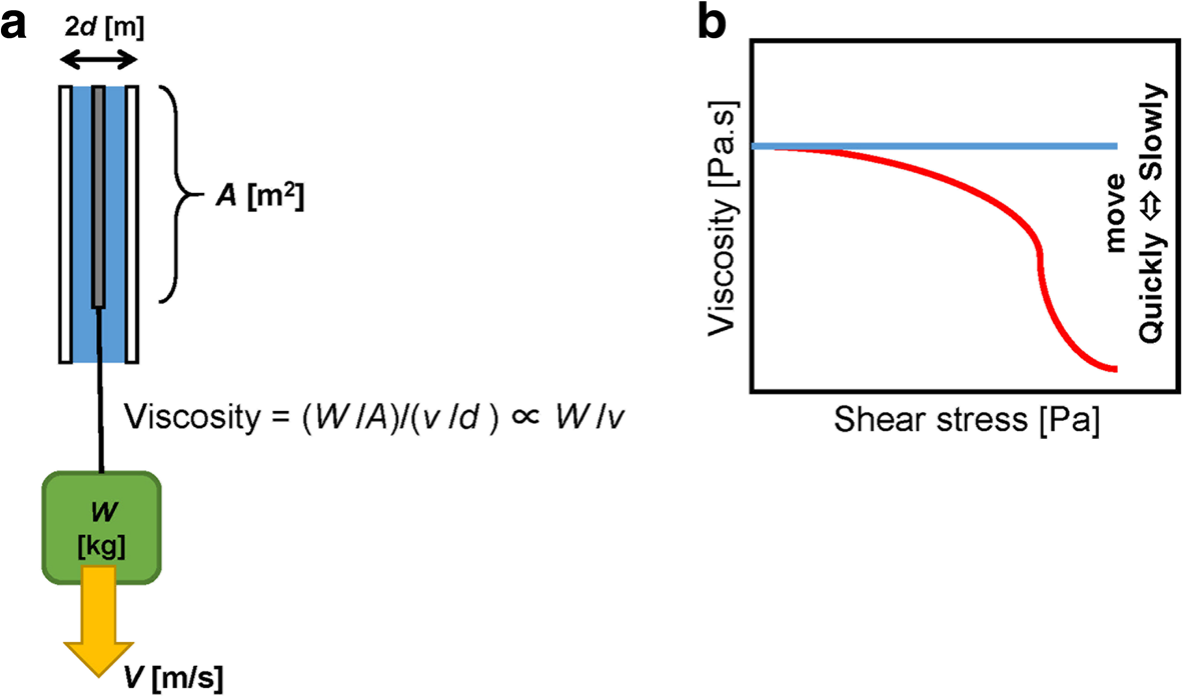
Meaning of viscosity. **a** Suppose that a thin plate (zero thickness) having an area A [m2] is sandwiched between the material of interest and large plates. They are aligned vertically, and a paper weight having a weight W [kg] is suspended from the thin plate. The paper weight falls at a speed v [m/s] when the distance between plates is 2d [m]. Viscosity is defined as follows: Viscosity = (*Wg/2A*)/(*v*/*d*) ∝ *W*/*v* (e.g., water has viscosity of 0.001 Pa·s). This equation indicates that the falling speed of the paper weight is proportional to the weight under constant viscosity. A heavier paper weight falls at a higher speed, and a lighter one falls more slowly. **b** If the viscosity is decreased with increasing shear stress, as expressed by the red curve, how does the paper weight falls? If the paper weight is sufficiently light, the falling speed is roughly same as that in the case of constant viscosity, blue line, because they have almost the same viscosity in the small shear stress region. In contrast, when a heavy paper weight is suspended, the paper weight must fall down quickly in the case of non-constant viscosity, because the falling speed is inversely proportional to viscosity. Therefore, a higher viscosity at a larger shear stress means that an obstacle stuck to the material moves more slowly. In other words, the obstacle feels a larger resistance in a more viscous material, and is difficult to move, which is characterized as being “more sticky”

Viscosity was measured with a stress-controlled rheometer (Anton-Paar Japan, Tokyo, Japan) on the following 8 materials: Dexi+Moist+Vase (DMV; i.e., admixture paste), Dexi, Dexi+Moist (DM), Moist, Moist+Vase (MV), Vase, cream-type denture adhesive New Poligrip® (GlaxoSmithKline K.K., Tokyo, Japan; hereinafter referred to as “Poli”) and cushion-type denture adhesive Toughgrip (Kobayashi Pharmaceutical Co., Ltd., Osaka, Japan; “Tough”). We prepared fresh DMV for each experiment. Viscosity *η* [Pa·s] and shear stress *σ* [Pa] were measured at two temperatures (25 °C and 37 °C to mimic room and oral temperatures, respectively) at shear rates (dγ/dt) varying from 0 to 30 s^− 1^. Originally, the method for viscosity measurement basically followed Japanese Industrial Standards (JISK7117–2). However, the aim of rheological measurement in the present study was to describe how the newly proposed material deforms with the application of force and to compare this with other well-known materials. In this research, we therefore measured viscosity at exponentially increasing shear stress levels. Figure 1 shows an explanation of viscosity and how it is plotted on a graph.

### Adhesive force measurement

**Fig. 2:**
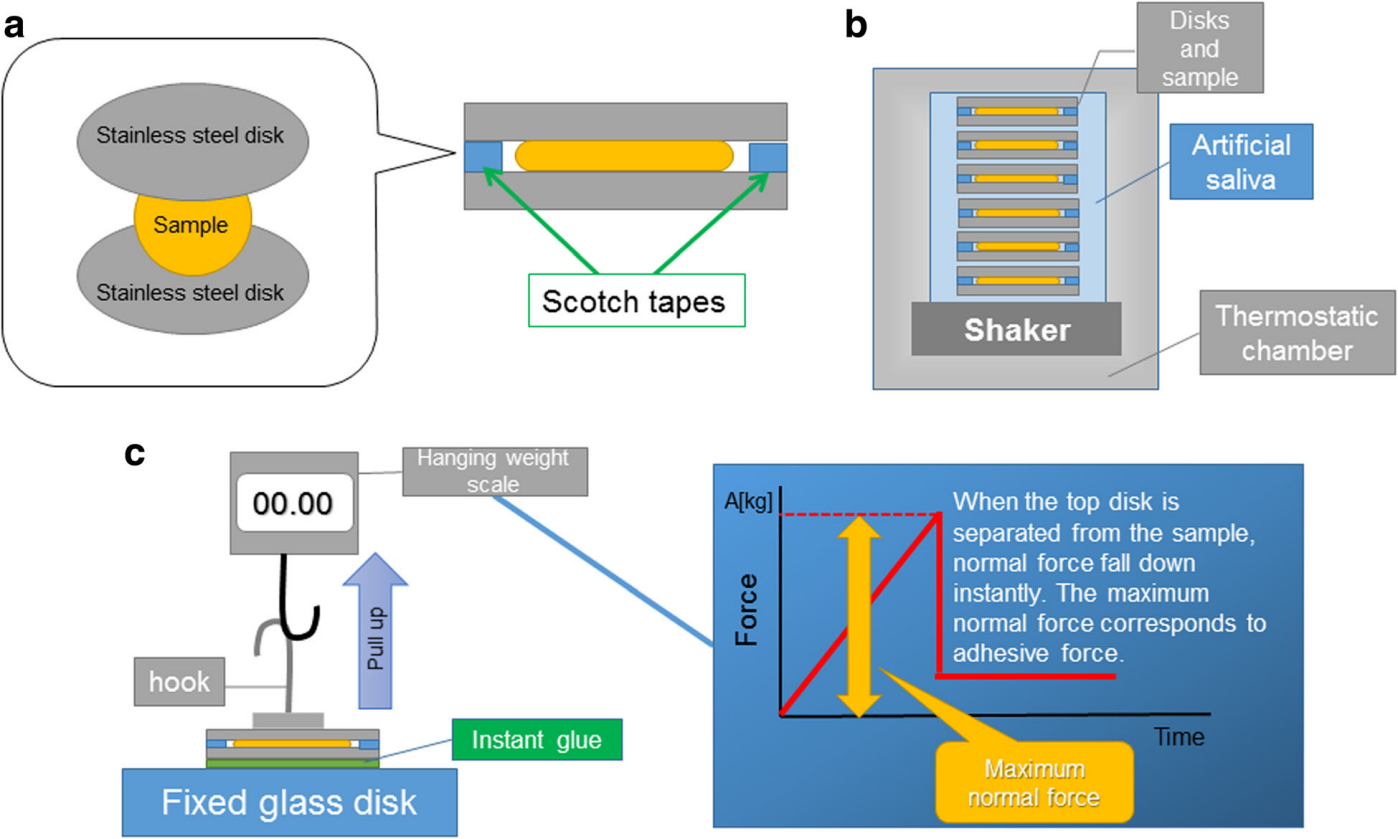
Adhesive force measurement **a**; Sample having a constant volume of 0.1 ml is squeezed between two stainless steel disks (diameter, 35 mm; thickness, 1 mm). Thickness of the sample between disks is maintained by 2 layers of scotch tape stuck on the bottom disk. **b**; Sample is immersed in artificial saliva, and shaken at 37 °C at 37 rpm (1, 2, 3, 4, 5 or 6 h). **c**; Bottom disk is firmly fixed to a glass disk, while a hook is fixed to the top plate using glue. A portable hanging weight scale is hitched to the hook on the top plate, and the hanging scale (see Additional File 2.) is pulled. Normal force caused by sample adhesiveness is measured

The measurement of adhesive force was performed on three types of sample, i.e., Poli, Tough and DMV, which were stored for up to 6 h in a simulated oral environment. Figure 2 shows the experimental procedure. Each sample having a roughly constant volume of 0.024 ml is squeezed between two stainless steel disks (SUS304; diameter, 35 mm; thickness, 1 mm). As two pieces of scotch tape are superposed on the bottom disk, the gap between the disks should be constant at 0.12 mm for all experiments. Subsequently, the cross-sectional area of the squeezed sample was constant at 200 mm^2^. The disks were then immersed in artificial saliva (Saliveht; Teijin Pharma Ltd., Tokyo, Japan) and incubated in an FMS-1000 thermostatic chamber (Tokyo Rikakikai Co., Ltd., Tokyo, Japan) at 37 °C while shaking at 37 rpm (MMS-3010; Tokyo Rikakikai Co., Ltd., Tokyo, Japan). Saliveht, which can only be prescribed in Japan, was used as artificial saliva throughout all experiments, and was treated with a Vacuum mixer (J. Morita Corporation, Tokyo, Japan) in order to remove CO_2_.

The bottom disk was firmly fixed onto a fixed glass disk using glue, while a hook was fixed on the top disk using strong double-side tape. A hanging weight scale (Electronic Portable Luggage Digital Scale, accuracy, 5 g; Weiheng, Shenzhen, China) was hitched to the hook attached to the top disk in order to measure the changes in normal force as a weight change. The normal force increased gradually as the top disk was stuck to the sample. However, the normal force instantaneously decreased when the top disk was separated from the sample. The hanging scale was manually pulled at a sufficiently slow and constant speed, such that the digits of the scale changed slowly to clearly see the maximum, and that the effect of acceleration on the measured value is negligible. The maximum normal force was determined from digit changes while pulling the scale. The adhesive force was divided by the cross-sectional area of the sample in order to account for the effects of variations in sample volume.

An additional movie file shows this experimental procedure in more detail (see Additional File 2).


**Additional file 2:** Adhesive force measurement. (3GP 772 kb)

$$ \mathrm{Adhesive}\ \mathrm{stress}\ \left[\mathrm{Pa}\right]=\frac{\mathrm{Adhesive}\ \mathrm{force}\ \left[\mathrm{kgf}\right]\cdot \mathrm{Gratational}\ \mathrm{acceleration}\ \left(=9{.801}^2/\mathrm{s}\right)}{\mathrm{Cross}-\mathrm{sectional}\ \mathrm{area}\ \mathrm{of}\ \mathrm{sample}\ \left[{\mathrm{m}}^2\right]} $$

In order to ensure the reproducibility of the adhesive force measurement, we prepared DMV samples three times, and repeated the measurement 15 times per each. The result indicates that we could prepare DMV with experimental error less than 10%, and the variation between samples was 50% of mean values. Since the variation was smaller than the difference of mean value between other two samples. Thus in this experiment, one sample was measured per condition.

### Elution tests under simulated oral environment

Each material was mixed with a blue water-soluble ink (THC-7C4N; Elecom, Osaka, Japan), kneaded and placed in a well of a 96-well tissue culture microplate (Iwaki, Tokyo, Japan) filled to a level that ensured all samples were at an equal volume. Each of the paste-filled wells was transferred to the wells of a 12-well tissue culture microplate (Iwaki), to which 5 ml of artificial saliva (Saliveht) was added. The above-mentioned micro-plate was used to prepare 6 sets, and was then placed on a PSU-2 T shaker (Waken B Tech Co., Ltd., Kyoto, Japan) and incubated in an FMS-1000 thermostatic chamber (Tokyo Rikakikai Co., Ltd., Tokyo, Japan) at 37 °C for 1–6 h while shaking at 37 rpm to test for elution of each material under conditions similar to the oral environment. Elution monitoring with a fixed-point camera was started immediately after addition of artificial saliva, and images were obtained after every hour.

Every hour, supernatant was collected from each well, and was transferred to a 96-well multiplate (200 μL per well). Absorbance at 535 nm was measured in triplicate for each sample in a SPECTRAmax (A) microplate reader (Molecular Devices Japan K.K., Tokyo, Japan). We performed measurements three times for each sample. Differences in the time course of elution between samples were assessed using a linear mixed-effects model with the main effects of time and group, and their interaction effect, as fixed effects, followed by post-hoc analyses to examine time course changes and sample differences. The time effect was treated as categorical. In addition, time to elution into saliva was compared among the samples using a log-rank test. Elution of the sample into saliva was defined as absorbance being more than 0.02, which was determined based on the absorbance of water-insoluble materials (cushion-type denture adhesive (Tough) and petrolatum (Vase)). All statistical analyses were performed using SPSS statistics version 22.0 software (IBM, Armonk, NY).

## Results

### Viscosity measurement

**Fig. 3:**
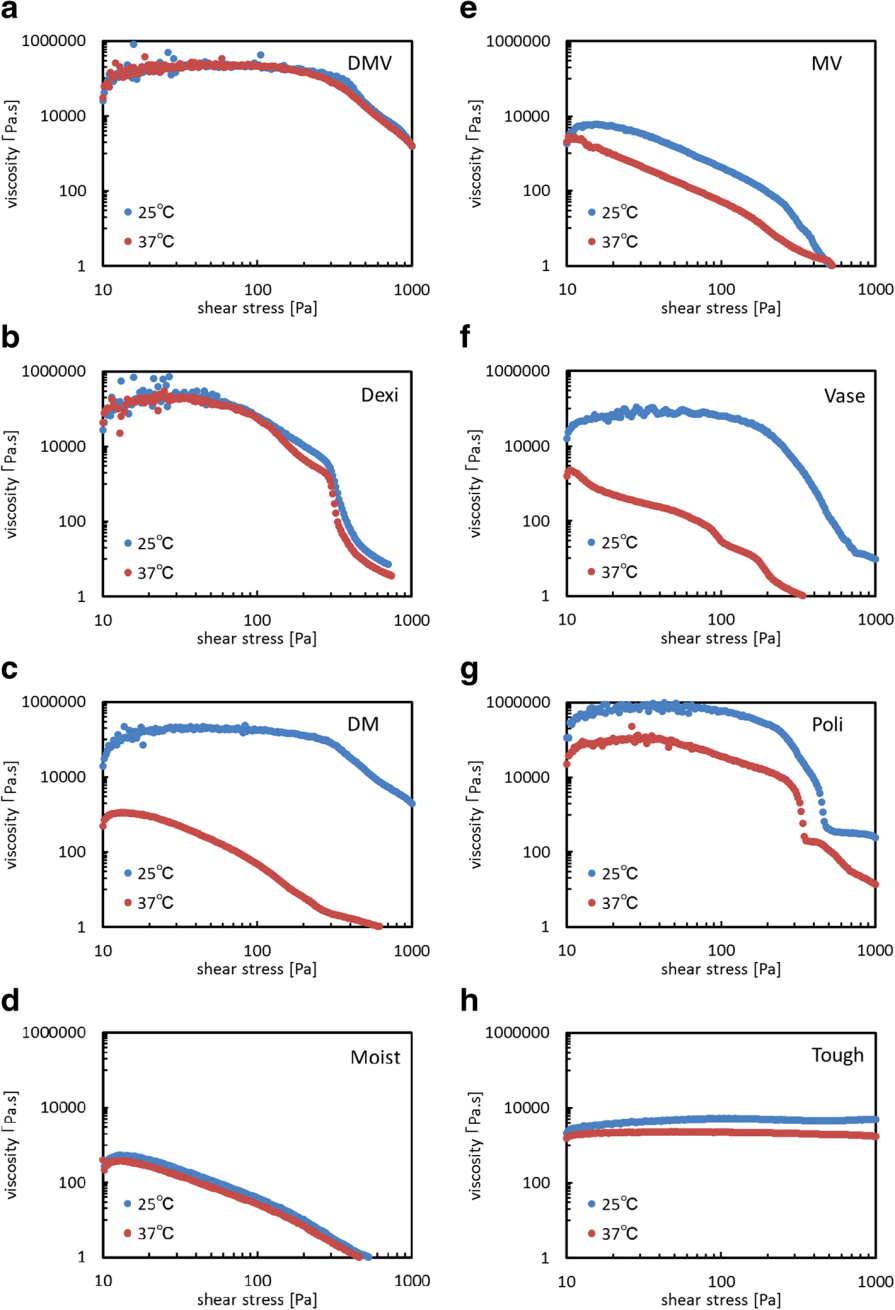
Viscosity of each material Horizontal and vertical axes represent shear stress [Pa] and shear viscosity [Pa·s], respectively, both on a logarithmic scale. Red and Blue dots represent data measured at 37 °C and 25 °C, respectively. Viscosity measurement was carried out using one sample of each substance. Figure 1 provides background information for the interpretation of this Fig. **a**) DMV: Mixed paste consisting of dexamethasone, gel for oral care and petrolatum. **b**) Dexi: Dexamethasone. **c**) DM: Mixed paste consisting of dexamethasone and gel for oral care. **d**) Moist: Gel for oral care. **e**) MV: Mixed paste consisting of gel for oral care and petrolatum. **f**) Vase: Petrolatum. **g**) Poli: Cream-type denture adhesive: New Poligrip®. **h**) Tough: Cushion-type denture adhesive: Toughgrip. This figure shows changes in the viscosity of each material when an increasing force was applied to them. All materials showed increasing fluidity with increasing force applied. More specifically, viscosity of “Tough” was small but constant while that of “Poli” was the highest and decreased with increasing stress and tempereature. “DMV” falls between them, showing relatively large viscosity even at the highest stresses, as well as no temperature dependence

The results of viscosity measurement are shown in Fig. 3. This graph shows the change of the relative difficulty of altering the shape of the sample with increasing force applied to each sample; viscosity increases with the values on the vertical axis.

DMV maintained a constant level of viscosity up to a certain level of stress, showing a stress-dependent pattern similar to that of Poli. However, both DMV and Poli showed marked decreases in viscosity under extreme stress. DMV showed a consistent level of viscosity at varying temperatures, as observed with Dexi, Moist and Tough, demonstrating its temperature independence. Tough showed neither stress nor temperature dependence, with its level of viscosity being slightly lower than those of the other materials and comparable to that of DMV under extreme stress. These findings indicate that DMV has combined properties of Poli and Tough. Furthermore, none of the components of DMV alone showed such properties. The timing of fluidity increases in DMV was almost the same as that in Poli.

### Adhesive force measurement

**Fig. 4:**
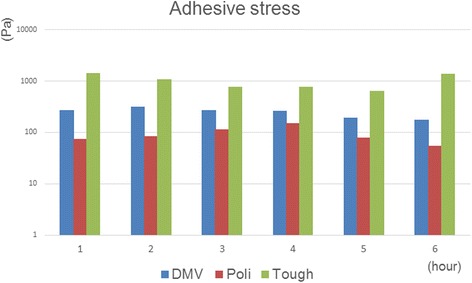
Adhesiveness force test. The results of measurement of adhesive force was performed on three kinds of samples, i.e., Poli, Tough and DMV, which are stored for up to 6 h in a simulated oral environment. Each bar expresses one sample (*n* = 1)

The results of adhesiveness tests are shown in Fig. 4. With regard to the 6 h temporal change, the temporal adhesion force changes for every sample were different, but the adhesive force maintained the same order of Tough>DMV > Poli. This suggests that the DMV showed stronger adhesiveness when compared with cream-type denture adhesives and weaker adhesiveness when compared with cushion-type denture adhesive in the oral environment. In the oral environment, we found that the adhesive stress of DMV falls between that of Poli and that of Tough, and this rank order (Tough>DMV > Poli) did not change after 6 h.

### Elution tests

**Fig. 5:**
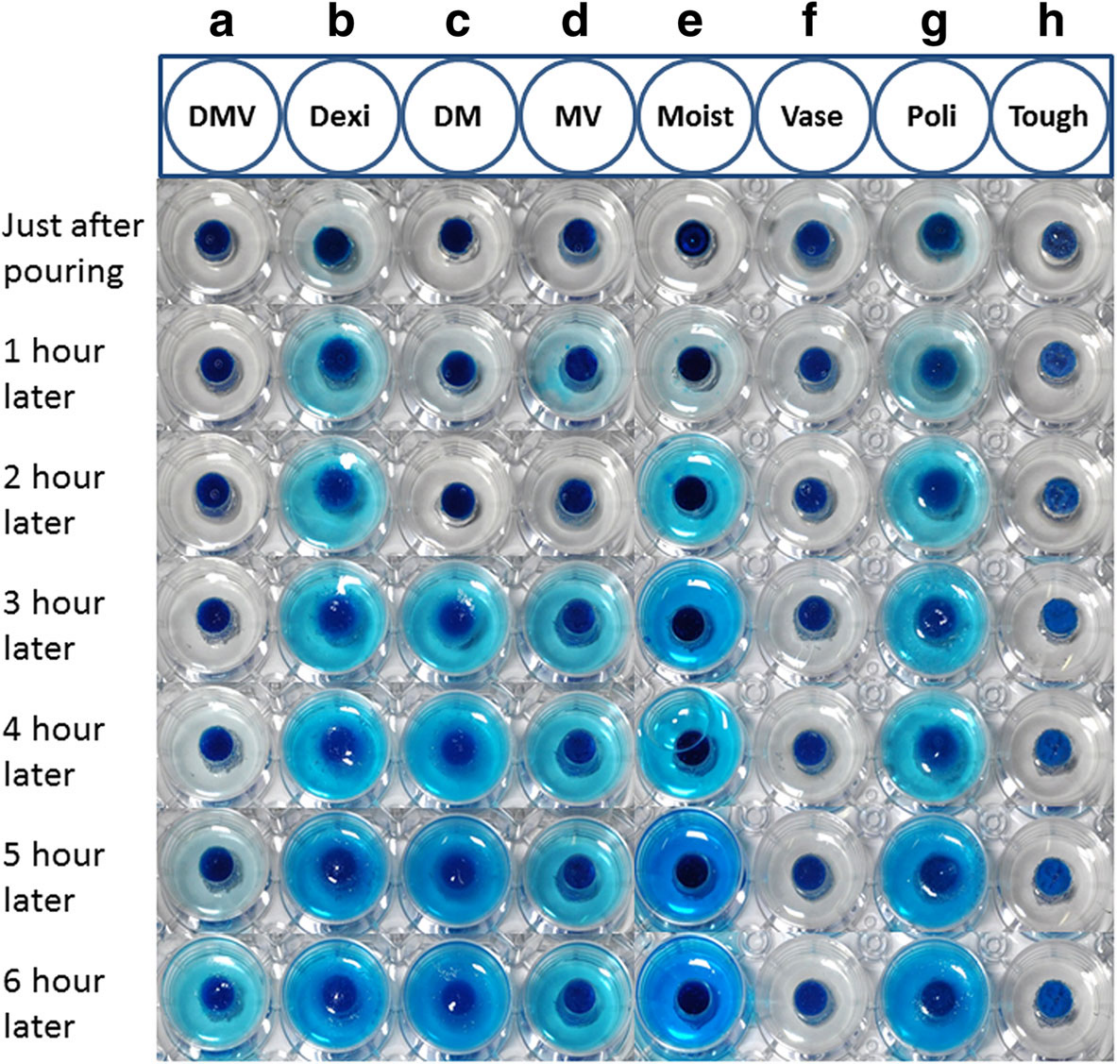
Elution monitoring. Elution monitoring immediately after the addition of distilled water, and at 1h, 2 , 3 , 4 , 5 or 6 h later. **a**) DMV: Mixed paste consisting of dexamethasone, gel for oral care and petrolatum. **b**) Dexi: Dexamethasone. **c**) DM: Mixed paste consisting of dexamethasone and gel for oral care. **d**) MV: Mixed paste consisting of gel for oral care and petrolatum. **e**) Moist: Gel for oral care. **f**) Vase: Petrolatum. **g**) Poli: Cream-type denture adhesive: New Poligrip® . **h**) Tough: Cushion-type denture adhesive: Toughgrip. Elution monitoring with a fixed-point camera was started immediately after the addition of artificial saliva. The figure shows elution monitoring immediately after the addition of artificial saliva, and at 1 h, 2 h, 3 h, 4 h, 5 h or 6 h later

**Fig. 6:**
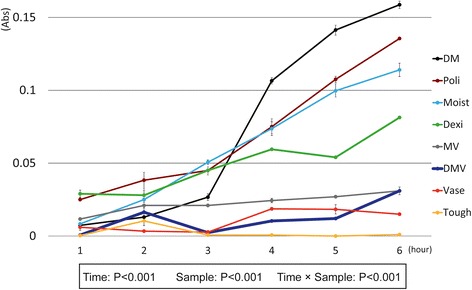
Absorbance measurement in paste elution test. Vertical axis represents absorbance measurements obtained with a spectrometer for each of the test materials arranged along the horizontal axis. Lower box shows the results for the linear mixed-effects model. Data are expressed as means ± S.D. (*n* = 3). Comparative materials are the same as those in Figs. 3 and 5

The results of the elution tests using the microplate setting are shown in Figs. 5 and 6. Linear mixed-effect model analysis demonstrated a significant difference in the time course of elution between the samples (*p*-value< 0.001 for interaction between time and group), the elution being increased in four samples (DM, Poli, Moist and Dexi), but not in the other four samples. The highest elution rate was observed for DM, followed in descending order by Poli, Moist, Dexi, MV, DMV, Vase and Tough. The elution of DMV was relatively stable. DMV showed similar temporal changes in elution as Vase (without significant differences between DMV and Vase). The log-rank test showed that there was a significant difference among the samples (*p* < .0001). This suggests that DMV gradually dissolves and remains firm for 6 h in the oral environment, and is comparable to paste with a greasy base. While no elution was observed with Tough, DMV maintained slight elution in artificial saliva for 6 h, suggesting that DMV has good moisture retention while maintaining the long-term steroid elution and helping to protect the affected area.

## Discussion

### Methodological considerations

The reasons for selecting the present components for the paste were as follows: steroid ointment is effective for pain relief in oral stomatitis [12, 13]; oral gel ameliorates the xerostomia occurring in head and neck cancer CRT [15, 16]; and Vaseline is a safe modifier for viscosity [17].

Admixture of the three agents (Dexi/Moist/Vase) yielded a mixed paste that was stickier than any single component or 2-component mixtures (Fig. 3), and this property did not show temperature dependence. These results may be explained as follows. Plastibase (Table 1), which is present in Dexi, is used to decrease temperature dependence (“Dexi”; Fig. 3B). On the other hand, although Dexi and Moist alone show temperature independence (Fig. 3B,D), temperature dependence was observed when mixing these two components (“DM”; Fig. 3C). It is possible that the sodium polyacrylate (superabsorbent polymer) present in Moist leads to temperature dependence, as it possesses hydrophilic and hydrophobic groups, causing it to desorb at high temperatures, thereby inducing viscosity changes [18].

The reason for the temporal change at 6 h is as follows: the interval between meals is about 6 h at most, as the majority of patients using an oral appliance would clean the oral appliance after each meal. As the mixed paste (DM, DV, MV and DMV) components were not quantitatively analyzed for subsidiary ingredients, we are uncertain whether such components exhibited effects on temperature dependence. However, the sodium polyacrylate present in Moist is associated with temperature changes, and when it was mixed with Dexi, viscosity might have changed with temperature.

Sodium polyacrylate is a type of superabsorbent polymer having a hydrophilic carboxyl group, and it forms a gel structure to incorporate a large number of water molecules into the meshwork [19, 20]. After mixing the three components, the balance of hydrophilic and hydrophobic groups changed, particularly due to the oil and fat components of the Vaseline, forming a gelatinous structure, which may have increased viscosity to a greater degree than expected.

The adhesiveness of the DMV was as stable as a commercial denture adhesive for 6 h. Based on the results in Fig. 4, we show that DMV has sufficient adhesive stress as a denture adhesive, and that DMV would be a useful denture adhesive. The DM samples mixed with gel-like structures were easily dissolved in artificial saliva (Fig. 6). On the other hand, when DM and Vase were mixed, the action of Vaseline’s hydrophobic groups was potent, and elution to artificial saliva of the DMV was significantly lower than DM. This suggests that DMV has long-term steroid efficacy and local retention in the oral environment. We cannot explain the reasons for the poor dissolution and persistent adhesiveness under a simulated oral environment in this paper. We believe that both the characteristics of sodium polyacrylate (containing Moist) and the osmotic pressure of artificial saliva (including NaCl) influenced our results. Miwa et al. reported that in artificial saliva, samples with a Vaseline base did not elute to saliva [21]. In addition, because the suction force of sodium polyacrylate, the gelling agent, included in Moist decreases on mixing with Dexi, temporal changes may have occurred. However, these physical properties require further investigation.

### Clinical application of admixture paste

In this paper, for a small number of patients, we applied admixture paste for the treatment of stomatitis. Subjective pain in DMV-treated patients was improved or unchanged, and slight macroscopic changes in stomatitis were seen. In addition, we have no data on a control group (patients without DMV-use) at this time, or sufficient data on the clinical use of DMV to confirm its clinical benefit. In Additional File 3, we show our clinical trial results to date, and we will present further results in the future.

Dexamethasone ointment is indicated for refractory stomatitis according to the package insert. In fact, steroid application is not typically the first-choice treatment for stomatitis. However, this admixture paste contains hinokitiol, the active ingredient in Refrecare H®, which has been shown to exert anti-microbial activity against *Candida* and to inhibit biofilm formation [22, 23].

The admixture paste has physical properties similar to those of denture adhesives and was developed with the intention of using it in denture-wearing patients. In patients with head and neck cancer, severe stomatitis may occur in both the oral cavity and in the pharynx. The latter often causes contact/swallowing pain and thereby prevents oral food intake [6, 24]. Denture use has been shown to be effective in improving mastication/swallowing disorder, increasing chewing-stimulated salivary secretion [25, 26] and preventing disuse atrophy of surrounding tissue in the head and neck [27]. The present admixture paste may therefore contribute to the maintenance of patient QOL by treating severe stomatitis and allowing them to use their oral appliances.

Meanwhile, the admixture paste contains dexamethasone, a steroid, and thus has a risk of inducing microbial substitution on long-term use. Its use should therefore be limited in patients with immune systems that have been compromised by CRT [12, 28]. At the same time, it has been shown to be highly effective in treating oral mucosal lesions, particularly for the relief of pain [29]. Dexamethasone is a steroidal agent and is generally recommended for use in aphthae or refractory stomatitis. Frequent monitoring of adverse reactions and worsening of symptoms by an oral surgeon is therefore recommended. As a future clinical theme, we believe that we need to examine the efficacy and safety of this admixture paste.

### Study limitations

There are some limitations in our research: an elution of admixture paste was exploratorily evaluated with no sample size calculation in advance; the adhesive force evaluation was performed with a single sample, which did not allow to make statistical comparisons and therefore compare differences. Thus, in the future, to demonstrate the hypothesis that admixture paste has an adhesiveness similar to that of denture adhesive and has gradual solubility, a behavioral study should be carried out over time, with a planned sample size.

## Conclusion

The application of admixture paste (mixture of dexamethasone, gel for oral care and petrolatum) for severe stomatitis in patients with head and neck cancer may facilitate oral food intake while using an oral appliance through its high levels of local retention, adhesiveness and gradual solubility in oral environment, which are not achieved with any of the 3 components alone.

## Additional files

Additional file 3: Clinical trial results to date. (DOCX 19 kb)

## Abbreviations

CRT: Radiotherapy and/or chemotherapy
Dexi: Dexamethasone
DM: Mixed paste consisting of dexamethasone and gel for oral care
DMV: Mixed paste consisting of dexamethasone, gel for oral care and petrolatum
Moist: Gel for oral care
MV: Mixed paste consisting of gel for oral care and petrolatum
PAP: Palatal augmentation prosthesis
Poli: Cream-type denture adhesive: New Poligrip®
QOL: Quality of life
Tough: Cushion-type denture adhesive: Toughgrip
Vase: Petrolatum
