# Supplementary material for: New paste for severe stomatitis in patients undergoing head-and-neck cancer radiotherapy and/or chemotherapy with oral appliance
Source: BMC Cancer. 2018 Mar 2;18:245. doi: 10.1186/s12885-018-4017-2 (PMC5834906; doi:10.1186/s12885-018-4017-2)
Supplement: Supplementary file 3 — Clinical trial results to date. (DOCX 19 kb) [file 12885_2018_4017_MOESM3_ESM.docx]

**〔Additional file 3〕**

**Simplified report of the Clinical application**

**1. Subjects**

Four patients who were hospitalized at the Hyogo College of Medicine Hospital for the treatment of head and neck cancer and received radiotherapy and/or chemotherapy were included in this study. While one patient received chemotherapy, another one received radiotherapy, and the remaining two received both chemo and radiotherapy. With regards to the use of an oral prosthesis, while two patients wore partial or complete dentures in both the maxilla and the mandible, the third one wore a denture only in the maxilla, and the fourth patient used a palatal augmentation prosthesis. The patients had grade 2 or 3 stomatitis according to of the Common Terminology Criteria for Adverse Events (CTCAE) v.4.0, (CTCAE v4.0; http://evs.nci.nih.gov/ftp1/CTCAE/, **Table A**).

**2. Usage and duration**

Since stomatitis in these patients was caused by radiotherapy and/or chemotherapy, at first, we performed denture adjustment and oral management practices such as oral cleaning, application of oral care gel, laser treatment and use of mouthwash containing a local anaesthetic. However, when the condition worsened in an obvious manner determined subjectively, and/or objectively, we applied an admixture paste (DMV) while continuing with the oral management course mentioned above. Patients provided written informed consent before application of DMV. The oral cavity was cleaned with a toothbrush and foam swab before application of DMV. Patients were instructed to use DMV after cleaning the oral cavity, similar to when they use denture adhesives. Two specialists in Oral and Maxillofacial Surgery visually inspected each patient's oral cavity for any adverse signs based on the standard stomatitis grading (CTCAE v4.0; **Table A**) every 2 days after application. The use of DMV was temporarily or permanently discontinued if stomatitis was resolved, or if the oral surgeons decided to change the treatment. However, we prohibited the application of DMV for more than 3 weeks, even if the stomatitis was not resolved.

**3. Therapy evaluation**

The response to treatment was evaluated both subjectively by patients and objectively according to the standard stomatitis grading. (In this additional file, we cannot show all the detailed results because it includes the future report)

**4．Clinical efficacy**

While three patients could easily consume food orally, one patient’s condition did not change objectively while using the oral appliances. However, all patients who used DMV reported relief from pain caused due to oral appliances. None of the patients complained of the taste and odour of DMV while eating. A secondary stomatitis infection did not occur in these four patients.

Two patients did not continue using DMV, even though an exacerbation of stomatitis was expected with radiotherapy. Since eating without the denture was impossible, the first patient strongly hoped to wear them during radiotherapy. Initially, we relined one denture with a soft lining material, but since it did not alleviate the pain enough, we started to use DMV. The pain was relieved during meals (modified diet) and the patient continued ingestion for 17 days though the food intake gradually decreased. After 50 Gy radiation, when the patient could not ingest anymore, tube feeding was started, and the use of DMV was stopped. In the second patient, both the upper and lower prostheses were attached to improve ingestion, deglutition and dysarthria, and to cover the vestibular dead space from a glossectomy reconstitution.　The patient continued ingestion with the prostheses until 40 Gy radiation was administered, which caused exacerbation of pharyngeal odynophagia. Ingestion was discontinued, and the use of DMV was unnecessary because tube feeding was started and irradiation under the use of prosthesis did not cause pain.

Based on the few case studies so far, it appears that application of DMV can improve stomatitis without causing secondary infections such as oral candidiasis. Clinical research is ongoing, and we will report the results elsewhere.

**Table A**. The diagnostic criteria for stomatitis

| Adverse Event | Grade 1 | Grade 2 | Grade 3 | Grade 4 | Grade 5 |
| --- | --- | --- | --- | --- | --- |
| Condition | Asymptomatic or mild symptoms;  intervention not indicated | Moderate pain not interfering  with oral intake;  modified diet indicated | Severe pain  interfering with oral intake | Life-threatening  Consequences; urgent  intervention indicated | Death |

We applied Common Terminology Criteria for Adverse Events (CTCAE) v4.0.12
